# Association between red blood cells transfusion and 28-day mortality rate in septic patients with concomitant chronic kidney disease

**DOI:** 10.1038/s41598-024-75643-3

**Published:** 2024-10-10

**Authors:** Lei Chen, Honglei Lu, Chenwei Lv, Haibin Ni, Renjun Yu, Bing Zhang, Xingxing Hu

**Affiliations:** 1 Third Clinical Medical College, Nanjing University of Traditional Chinese Medicine, Nanjing, Jiangsu China; 2 Department of Intensive Care Medicine, Affiliated Hospital of Integrated Traditional Chinese and Western Medicine, Nanjing University of Chinese Medicine, Nanjing, Jiangsu China; 3 Department of Intensive Care Medicine, Jiangsu Province Academy of Traditional Chinese Medicine, Nanjing, Jiangsu China; 4 Department of Emergency, NanJing LiShui District Hospital of Traditional Chinese Medicine, Nanjing, Jiangsu China

**Keywords:** Intensive care unit, Sepsis, CKD, Red blood cell, MIMIC-IV, Chronic kidney disease, Medical research

## Abstract

Patients with chronic kidney disease (CKD) often have impaired immune function, making them more prone to infections that can lead to sepsis. The coexistence of these conditions can result in decreased hemoglobin levels and is associated with a higher mortality rate. To investigate whether the transfusion of red blood cells (RBCs) improves the prognosis of septic patients with concomitant CKD and to explore the indications for red blood cell transfusion. This retrospective cohort study utilizes data from the MIMIC-IV (v2.0) database. The study enrolled 6,604 patients with sepsis and concomitant CKD admitted to the Intensive Care Unit (ICU). Propensity score matching (PSM) was applied to adjust for confounding factors. Multivariate Cox regression analysis revealed an association between RBC transfusion and a decreased risk of 28-day mortality (HR: 0.61, 95% CI: 0.54–0.70, *P* < 0.001). Following a meticulous 1:1 propensity score matching analysis between the two cohorts, the matched population revealed a notable decrease in 28-day mortality within the RBC transfusion group (HR: 0.60, 95% CI: 0.51–0.71; *P* < 0.001). Additionally, we observed that a SOFA score ≥ 5, a Base Excess (BE) value < 3, and an estimated Glomerular Filtration Rate (eGFR) < 30 may be considered when evaluating the potential need for RBC transfusion. This study demonstrated an association between RBC transfusion and decreased 28-day mortality in patients with sepsis accompanied by CKD. The patient’s BE value, SOFA score, and eGFR are crucial factors influencing the treatment outcome and should be considered when deciding on RBC transfusion.

**Supplementary Information:**

The online version contains supplementary material available at 10.1038/s41598-024-75643-3.

## Introduction

Sepsis is a systemic inflammatory response syndrome caused by infection and is characterized by an exaggerated inflammatory reaction to infection that leads to organ dysfunction^1^. Chronic kidney disease (CKD) constitutes a pervasive global health challenge, manifesting with a prevalence ranging from 10 to 16% worldwide, thereby substantially affecting the quality of life for afflicted individuals^2,3^. Individuals with CKD frequently exhibit compromised immune function, potentially heightening their susceptibility to sepsis, consequently leading to increased rates of unfavorable renal events and elevated mortality^4,5^. Research indicates that CKD patients with a lower estimated glomerular filtration rate(eGFR) have a greater risk of infection than those with a higher eGFR^6,7^. The coexistence of sepsis and persistent renal dysfunction in patients is thought to be linked to an elevated risk of mortality^8^.

Sepsis and CKD are both associated with a decline in hemoglobin levels^9^. Although the etiology of CKD is multifaceted, EPO deficiency may be the primary contributor to anemia in CKD patients^10^. Anemia in septic patients is predominantly linked to increased red blood cell destruction or reduced synthesis due to systemic inflammatory reactions^11^. Furthermore, factors such as iatrogenic blood loss, hemodilution, nutritional deficiencies, and specific medication usage can also reduce patients’ hemoglobin levels^12,13^. Studies suggest that anemic status is correlated with deteriorating renal function, heightened risk of acute hospitalization, and increased mortality in such patients^14–16^. Erythropoiesis-stimulating Agents (ESA) can stimulate EPO production, elevate hemoglobin levels, and ameliorate anemia, representing a primary therapeutic approach for addressing low hemoglobin levels in CKD patients^17^. However, in septic patients, the excessive and uncontrolled release of pro-inflammatory mediators can lead to ESA hypo-responsiveness, consequently limiting the efficacy of other medications^18,19^. Therefore, for this specific population of patients with sepsis and concurrent CKD, red blood cell (RBC) transfusion may be a suitable intervention. Although RBC transfusion has been shown in various studies to be associated with adverse reactions such as blood volume overload, hyperkalemia, iron overload, transfusion-related infections, fever, and alloimmunization^20^, there is currently a shortage of clinical trials investigating the impact of RBC transfusion on outcomes in patients with sepsis and CKD.

While certain earlier studies proposed the appropriateness of a restrictive RBC transfusion strategy for septic patients^21,22^, these investigations were limited by the small cohort of individuals at risk of anemia. Consequently, the optimal transfusion threshold for RBC in patients with sepsis and concurrent CKD remains to be determined. Moreover, there has yet to be a thorough exploration of whether clinical variables other than hemoglobin concentration can serve as indications for RBC transfusion.

The aim of this study was to examine the influence of RBC transfusion on the prognosis of patients with sepsis and CKD and to elucidate potential indications for RBC transfusion.

## Materials and methods

### Study subjects

**Fig. 1:**
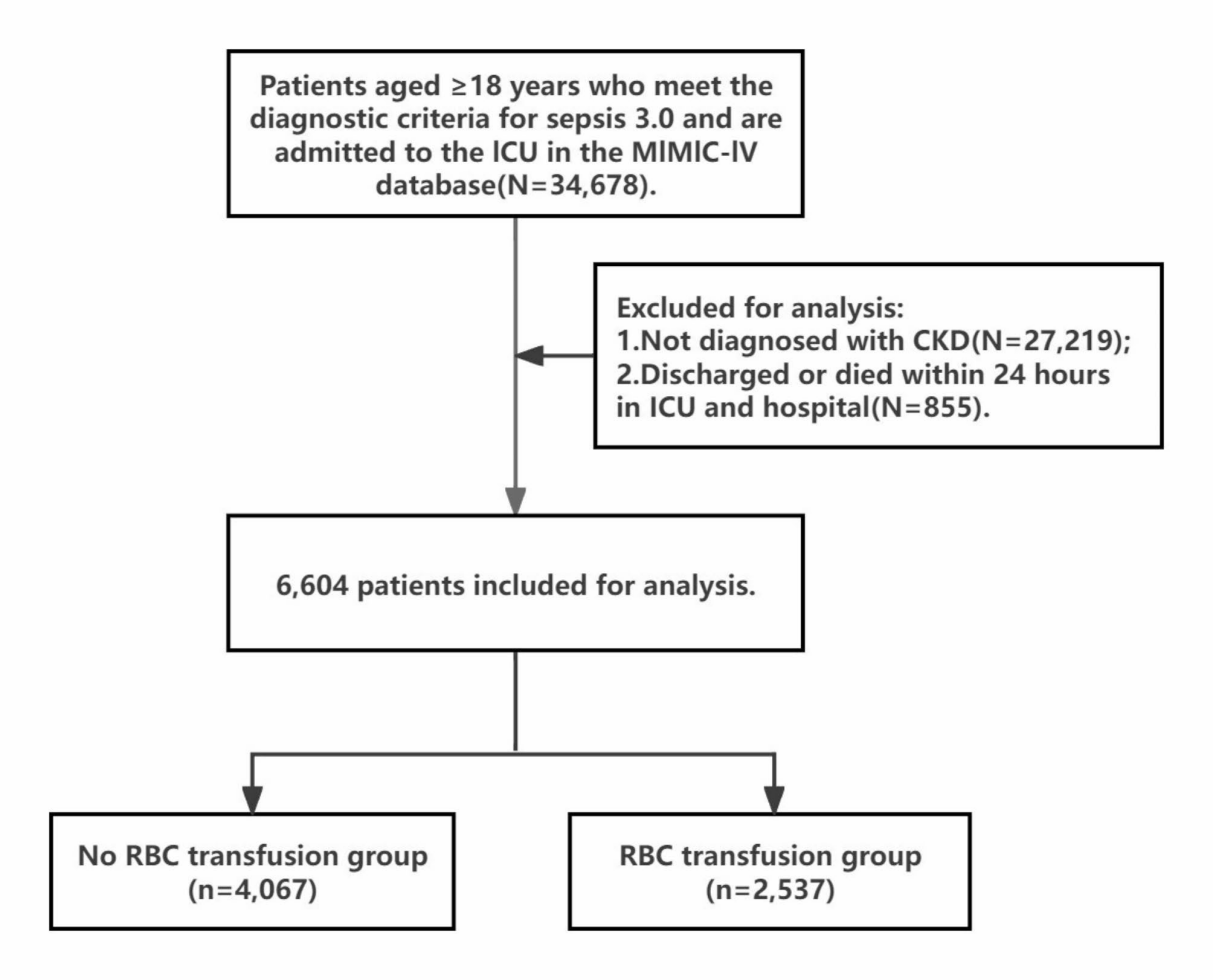
Study flowchart.

The data for this research were obtained from the Medical Information Mart for Intensive Care IV (MIMIC-IV) database, which serves as a freely accessible critical care medical dataset. The MIMIC-IV is a collaborative initiative involving the Massachusetts Institute of Technology (MIT) Laboratory for Computational Physiology, Beth Israel Deaconess Medical Center (BIDMC), and Philips Healthcare. The dataset encompasses clinical data from hospitalized patients at the Beth Israel Deaconess Medical Center between 2013 and 2019^23^. The author of this article has been granted authorization (Record ID: 52921014) to access and use the database for this study. A total of 6604 patients were included in the research present study. Eligible participants met the following criteria: (1) patients aged 18 years or older and (2) critically ill adult patients with chronic renal disease (CKD) who met the Sepsis 3.0 criteria^1^. The criteria for diagnosing CKD include either of the following being present for more than three months: (1) Markers of kidney damage, such as albuminuria (AER ≥ 30 mg/24 hours or ACR ≥ 30 mg/g), urine sediment abnormalities, electrolyte and other abnormalities due to tubular disorders, abnormalities detected by histology, structural abnormalities detected by imaging, or a history of kidney transplantation; (2) A decreased glomerular filtration rate (GFR) of less than 60 mL/min/1.73 m²^24^. Patients who were discharged or deceased within 24 h (*n* = 885) were excluded from the analysis. Furthermore, we only analyzed patients admitted to the ICU for the first time (Fig. 1).

## Data extraction

We utilized PostgreSQL (version 15.0) and Navicat Premium (version 16.0) software to extract variables from the MIMIC-IV database. Variables with missing values ≥ 40% were excluded from the analysis, while the remaining variables were subjected to the k-nearest neighbor algorithm to address the missing data. We extracted the admission hemoglobin concentration, the lowest hemoglobin levels within 48 h after admission to the ICU, and the volume of red blood cell transfusion, as well as the following datasets: (1) Demographic information: age, sex, ICU type. (2) Vital signs: heart rate(HR), respiration rate(RR), systolic blood pressure(SBP), diastolic blood pressure(DBP), mean arterial pressure(MAP) and blood oxygen saturation(SpO_2_). (3) Laboratory indicators: white blood cell(WBC), platelets, Bilirbin, aspartate transaminase(AST), alanine transaminase(ALT), urea nitrogen(BUN), creatinine, pH, PaO_2_, PaCO_2_, lactate, bicarbonate, base excess(BE), sodium, potassium and chlorine levels. (4) Comorbidities: anemia. (5) Severity of illness score: SOFA score, APS III score, OASIS score, SAPS II score, SIRS score, and GCS score. (6) Erythropoiesis-stimulating agents (ESAs), iron preparations, norepinephrine, vasopressin, phenylephrine, and epinephrine were used, along with ventilation and renal replacement therapy (RRT). The primary outcome was 28-day mortality. Secondary outcomes included one-year mortality, ICU length of stay, and hospital length of stay.

### Statistical analysis

Based on whether patients received RBC transfusion within 48 h after admission to the ICU, we divided the study cohort into an intervention group (RBC transfusion group) and a control group (No RBC transfusion group). Continuous variables with a normal distribution are represented as the mean(standard deviation), while nonnormally distributed continuous variables are presented as the median(interquartile range). Between-group comparisons were performed using paired t-tests or Wilcoxon rank-sum tests. Categorical variables are expressed as percentages and were compared between groups using the χ^2^ test.

Cox multivariate regression analysis was examined the association between the 28-day mortality rate and RBC transfusion. Patient survival and clinical characteristics will be assessed utilizing Kaplan‒Meier curves, with comparisons conducted using the log-rank test. Then, to reduce the impact of potential confounders, we employed propensity score matching(PSM). The propensity scores were calculated using logistic regression, considering the aforementioned demographic and clinical characteristics. We assessed the covariate balance before and after matching using absolute standardized mean differences (SMDs) and specified an SMD greater than 0.1 as a relevant imbalance^25^. We performed 1:1 greedy nearest neighbor matching with a caliper of 0.01. Additionally, to ascertain whether hemoglobin levels influence the therapeutic efficacy of red blood cell transfusion, we constructed a model that includes the interaction term between the lowest hemoglobin levels and RBC transfusion, aiming to evaluate the interaction between hemoglobin levels and RBC transfusion.

Finally, subgroup analyses were performed to validate the impact of RBC transfusion on the 28-day mortality rate within specific participant subsets, categorized by age, MAP, lactate, BE, SOFA score, ESA, norepinephrine, vasopressin, ventilation, RRT, anemia and eGFR^26^. All the statistical analyses were performed using R software (version 4.2.2), with the significance level set at *P* < 0.05.

## Results

### Baseline characteristics

The severity of the RBC transfusion group’s condition was notably more pronounced than that of the No RBC transfusion group. The former demonstrated a significantly lower hemoglobin level [8.50 (7.50, 9.80) vs. 10.40 (9.10, 11.80)] and a higher SOFA score upon admission in contrast to the latter [8.0 (6.0, 12.0) vs. 6.0 (5.0, 9.0)]. Within the initial 24 h of ICU admission, a more significant proportion of patients in the RBC transfusion group underwent treatment with norepinephrine (29% vs. 45%), mechanical ventilation (34% vs. 62%), and renal replacement therapy (29% vs. 40%)(Table S1). Following the implementation of 1:1 propensity score matching, a total of 2,582 patients were enrolled in the matched cohort, with 1,291 patients in each group. This matching process was employed to create comparable groups and reduce the impact of potential confounding factors by ensuring that each patient in the RBC transfusion group was paired with a patient in the No RBC transfusion group with similar baseline characteristics. As a result, the imbalance of variables was significantly reduced, with all variables achieving an absolute standardized mean difference (SMD) of less than 0.1, indicating well-balanced groups. In the matched cohort, 780 patients (30.2%) were admitted to the medical ICU, representing the most substantial portion of ICU admissions. Moreover, 1,268 patients (49.1%) required mechanical ventilation support at the time of ICU admission, and 872 patients (33.8%) required renal replacement therapy (Table 1).

**Table 1:** Baseline covariates before and after matching.

Variables	Before Matching			After Matching		
	No RBC transfusion group (*n* = 4,067)	RBC transfusion group (*n* = 2,537)	SMD	No RBC transfusion group (*n* = 1,291)	RBC transfusion group (*n* = 1,291)	SMD
Age, years	72.37 (13.66)	70.13 (13.61)	-0.165	71.27 (13.26)	71.56 (13.47)	0.021
Sex, No.(%)						
Male	2470 (60.7)	1548 (61.0)	0.006	768 (60.8)	770 (60.9)	0.003
Female	1597 (39.3)	989 (39.0)	-0.006	496 (39.2)	494 (39.1)	-0.003
ICU type, No.(%)						
MICU	1383 (34.0)	677 (26.7)	-0.166	381 (30.1)	372 (29.4)	-0.016
SICU	440 (10.8)	353 (13.9)	0.089	155 (12.3)	157 (12.4)	0.005
CCU	670 (16.5)	288 (11.4)	-0.161	176 (13.9)	174 (13.8)	-0.005
TICU	427 (10.5)	589 (23.2)	0.301	236 (18.7)	240 (19.0)	0.007
Other	1147 (28.2)	630 (24.8)	-0.078	316 (25.0)	321 (25.4)	0.009
HR, beats/min	88.40 (20.35)	88.52 (19.61)	0.007	87.35 (19.98)	87.30 (19.54)	-0.003
SBP, mmhg	123.78 (26.76)	119.47 (27.22)	-0.158	121.52 (26.39)	120.60 (27.71)	-0.034
DBP, mmhg	65.90 (18.92)	61.81 (19.02)	-0.215	63.47 (18.81)	62.66 (19.76)	-0.042
MAP, mmhg	85.19 (18.95)	81.03 (19.34)	-0.215	82.82 (18.72)	81.98 (19.91)	-0.044
RR, beats/min	20.13 (6.07)	19.31 (5.94)	-0.138	19.25 (5.72)	19.46 (5.95)	0.036
Temperature,℃	36.71 (0.89)	36.55 (0.97)	-0.163	36.59 (0.98)	36.60 (0.89)	0.009
SPO_2_,(%)	96.43 (4.23)	97.15 (4.58)	0.157	97.06 (4.15)	97.06 (4.82)	0.001
WBC,10^9/L	12.66 (9.98)	13.09 (13.98)	0.030	12.55 (9.73)	12.52 (8.85)	-0.002
Platelets,10^9/L	216.08 (109.37)	201.23 (122.55)	-0.121	209.15 (113.92)	210.61 (117.86)	0.012
Hemoglobin, g/dL	10.59 (1.96)	8.75 (1.88)	-0.983	9.26 (1.51)	9.23 (1.89)	-0.014
Lowest hemoglobin levels, g/dL	9.14 (1.67)	7.22 (1.14)	-1.680	7.79 (1.09)	7.72 (1.18)	-0.058
Bilirubin, mg/dL	1.13 (2.56)	1.73 (4.02)	0.150	1.42 (3.83)	1.45 (3.47)	0.008
AST, U/L	112.81 (453.01)	136.70 (552.70)	0.043	121.96 (513.79)	126.92 (462.14)	0.009
ALT, U/L	73.85 (263.79)	79.24 (301.37)	0.018	76.32 (246.92)	79.13 (301.94)	0.009
Creatinine, mg/dL	3.23 (2.58)	3.19 (2.54)	-0.017	3.14 (2.52)	3.17 (2.52)	0.014
BUN, mmol/L	46.86 (27.96)	51.22 (32.12)	0.136	48.78 (30.76)	49.23 (30.05)	0.014
pH	7.34 (0.10)	7.35 (0.11)	0.037	7.35 (0.10)	7.35 (0.10)	-0.009
PaO_2_,mmhg	121.45 (107.69)	159.09 (128.61)	0.293	149.97 (131.10)	148.81 (122.46)	-0.009
PaCO_2_,mmhg	45.48 (14.27)	42.61 (12.79)	-0.225	43.26 (13.29)	43.59 (13.40)	0.026
Bicarbonate, mmol/L	22.86 (5.57)	21.89 (5.44)	-0.179	22.25 (5.55)	22.29 (5.35)	0.008
BE, mmol/L	-1.52 (8.35)	-2.15 (5.78)	-0.109	-1.77 (5.65)	-1.83 (5.43)	-0.011
Lactate, mmol/L	2.23 (1.69)	2.32 (2.13)	0.043	2.18 (1.84)	2.18 (1.84)	0.000
Potassium, mmol/L	4.66 (1.05)	4.58 (0.94)	-0.076	4.56 (0.99)	4.57 (0.92)	0.015
Sodium, mmol/L	137.69 (5.93)	137.75 (5.50)	0.011	137.79 (5.71)	137.70 (5.43)	-0.016
Chlorine, mmol/L	101.00 (7.68)	102.61 (7.65)	0.211	102.34 (7.77)	102.25 (7.37)	-0.012
SOFA score	6.99 (3.33)	8.84 (3.93)	0.469	7.84 (3.43)	7.86 (3.54)	0.006
SIRS score	2.60 (0.91)	2.77 (0.91)	0.181	2.70 (0.91)	2.69 (0.92)	-0.014
OASIS score	34.29 (9.02)	37.49 (9.67)	0.331	36.17 (9.25)	35.92 (9.18)	-0.026
APSIII score	59.97 (20.69)	69.74 (25.18)	0.388	64.65 (23.24)	63.98 (21.85)	-0.026
SAPSII score	42.96 (12.62)	46.97 (13.64)	0.294	44.84 (12.98)	45.13 (13.03)	0.021
GCS score	14.21 (1.99)	14.28 (2.05)	0.034	14.31 (1.95)	14.29 (2.01)	-0.010
ESA, No.(%)	564 (13.9)	468 (18.4)	0.118	200 (15.8)	215 (17.0)	0.031
Iron preparation, No.(%)	516 (12.7)	370 (14.6)	0.054	193 (15.3)	203 (16.1)	0.022
Norepinephrine, No.(%)	1172 (28.8)	1151 (45.4)	0.332	466 (36.9)	469 (37.1)	0.005
Vasopressin, No.(%)	291 (7.2)	537 (21.2)	0.343	159 (12.6)	167 (13.2)	0.015
Phenylephrine, No.(%)	751 (18.5)	954 (37.6)	0.395	384 (30.4)	387 (30.6)	0.005
Epinephrine, No.(%)	137 (3.4)	311 (12.3)	0.271	89 (7.0)	92 (7.3)	0.007
Ventilation, No.(%)	1401 (34.4)	1568 (61.8)	0.563	650 (51.4)	631 (49.9)	-0.031
RRT, No.(%)	1162 (28.6)	1017 (40.1)	0.235	408 (32.3)	419 (33.1)	0.018
Anemia, No.(%)	1688 (41.5)	993 (39.1)	-0.048	544 (43.0)	549 (43.4)	0.008
eGFR, ml/min/1.73m^2^	29.96 (20.57)	30.95 (21.89)	0.045	31.10 (21.72)	30.92 (21.73)	-0.008

SMD Standardized Mean Difference, HR Heart rate, SBP Systolic blood pressure, DBP Diastolic blood pressure, MAP Mean arterial pressure, RR Respiratory rate, SpO_2_ Percutaneous oxygen saturation, WBC White blood cell, Lowest hemoglobin levels the lowest hemoglobin levels within 48 h after admission to the ICU, AST Aspartate aminotransferase, ALT Alanine aminotransferase, BUN Blood urea nitrogen, PaO_2_ Arterial oxygen pressure, PaCO_2_ Arterial carbon dioxide pressure, BE Base excess, SOFA score Sequential organ failure assessment score, SIRS score Systemic inflammatory response syndrome score, OASIS score Oxford acute severity of illness score, APSIII score Acute physiology score III, SAPSII score Simplified acute physiology score II, GCS score Glasgow coma score, ESA Erythropoiesis-stimulating agent, RRT Renal replacement therapy, eGFR estimated glomerular filtration rate.

### Association of RBC transfusion with outcomes

**Fig. 2:**
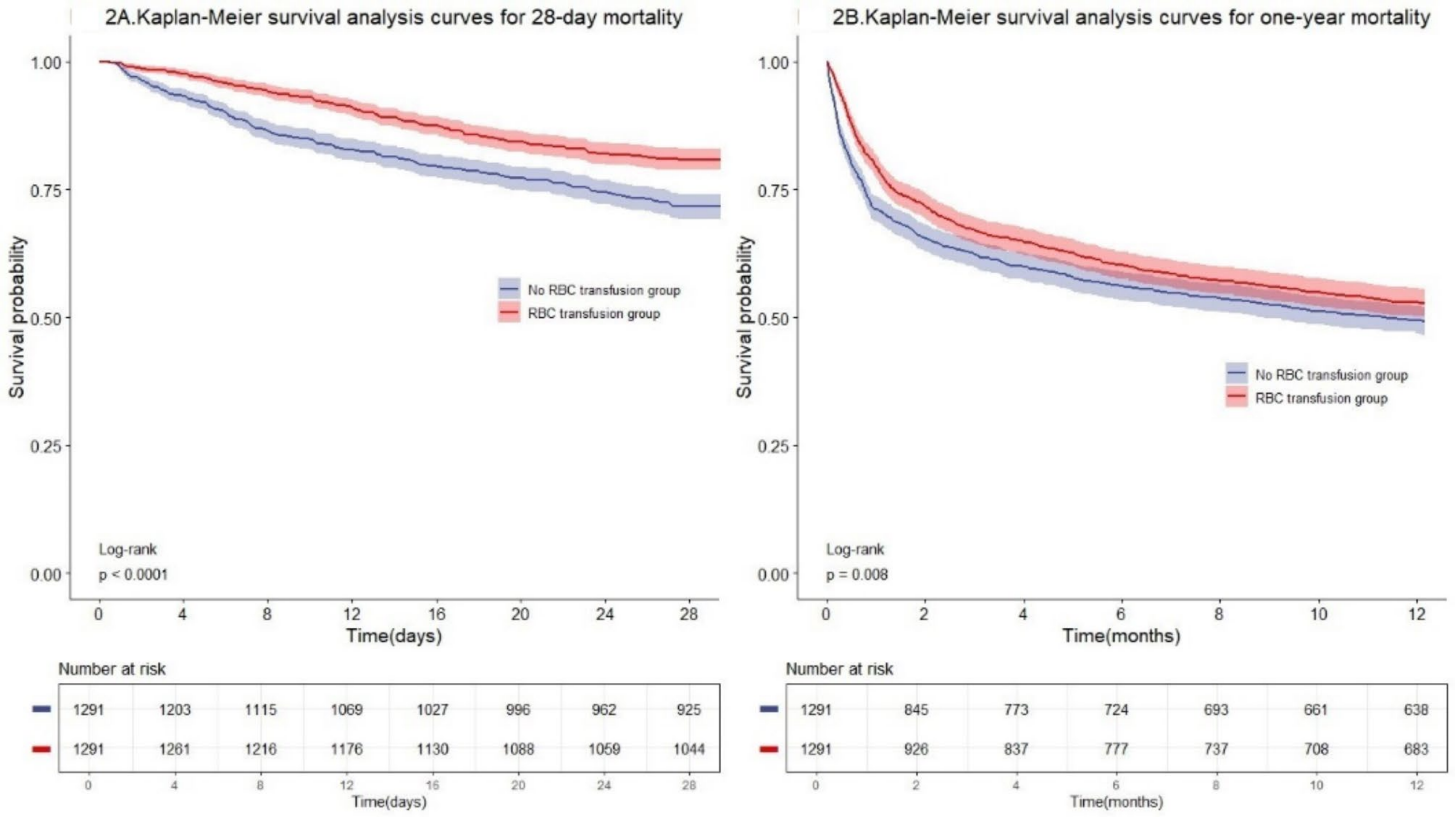
Kaplan-Meier Survival Plot for RBC transfusion after PSM. X-Axis: Survival time (days). Y-Axis: Survival probability.

The Kaplan-Meier survival curve showed that red blood cell transfusion patients exhibited significantly lower all-cause mortality at 28 days (log-rank test: *P* = 0.021)(Fig S1). After propensity score matching, patients in the RBC transfusion group had a longer median (IQR) LOS in the ICU [4.3 (2.3, 8.8) days vs. 3.0 (1.8, 5.1) days; *P* < 0.001] and in the hospital [12 (7, 21) days vs. 10 (6, 17) days; *P* < 0.001] (Table 2). However, the 28-day mortality rate in the RBC transfusion group was notably lower than that in the No RBC transfusion group[247 cases(19.1%) vs. 366 cases((28.4%); *P* < 0.001], with an HR of 0.63 (95% CI: 0.53, 0.74). The one-year mortality rate in the RBC transfusion group was also reduced [610 cases (47.3%) vs. 656 cases (50.8%); *P* = 0.008], yielding an HR of 0.86 (95% CI: 0.77, 0.96)] (Table 2). The Kaplan-Meier survival curve also demonstrates a significantly higher survival rate among patients in the RBC transfusion group(Fig. 2).

**Table 2:** Outcome analyses between two groups.

Characteristic	No RBC transfusion group	RBC transfusion group	HR	95% CI	*p*-value
Total matched cohort, No.	1,291	1,291			
ICU mortality, [n (%)]	216 (16.7%)	157 (12.2%)	0.68	0.56, 0.84	< 0.001
28-day mortality, [n (%)]	366 (28.4%)	247 (19.1%)	0.63	0.53, 0.74	< 0.001
One-year mortality, [n (%)]	656 (50.8%)	610 (47.3%)	0.86	0.77, 0.96	0.008
LOS ICU	3.0 (1.8, 5.1)	4.3 (2.3, 8.8)			< 0.001
LOS hospital	10 (6, 17)	12 (7, 21)			< 0.001

HR Hazard Ratio, CI Confidence Interval, LOS ICU Length of stay in intensive care unit, LOS hospital Length of stay in hospital.

Multivariate Cox regression analysis revealed a reduced 28-day mortality rate among septic patients with concomitant CKD following RBC transfusion (HR:0.61, 95%CI:0.54, 0.70, *P* < 0.001) (Table S2 ). After propensity score matching analysis, the matched population analysis demonstrated significant benefits for patients who received RBC transfusion (HR:0.60, 95%CI:0.51, 0.71, *P* < 0.001) (Table S3).

## Influence of hemoglobin on the treatment effect of RBC transfusion

**Fig. 3:**
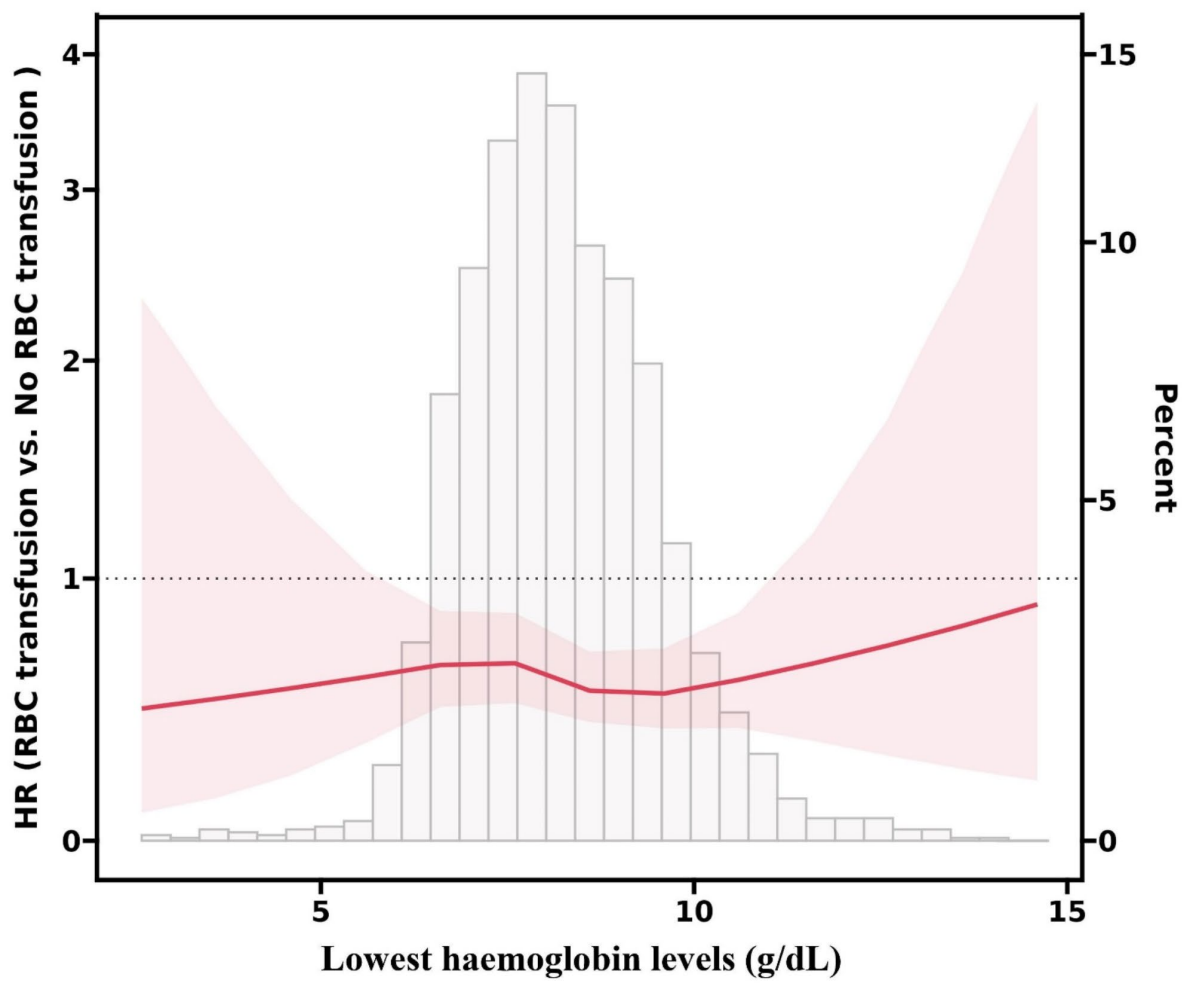
Spline figures plotting HR with RBC transfusion compared with no RBC transfusion for all-cause death based on lowest hemoglobin levels. Model with 4 knots located at the 5th, 35th, 65th and 95th percentiles.

Based on the restricted cubic spline plots (Fig. 3), the hazard ratio (HR) for 28-day mortality remains relatively stable across varying levels of the lowest hemoglobin, indicating that RBC transfusion’s impact on mortality does not significantly differ based on hemoglobin levels (P for interaction = 0.949).

Table 3 further supports this finding, showing that RBC transfusion is consistently associated with reduced 28-day mortality across all hemoglobin subgroups, with HRs below 1 in each category. The consistent HR across subgroups suggests that the effect of RBC transfusion on mortality is independent of the specific hemoglobin level.

**Table 3:** Threshold analysis (Cox Model).

Subgroup	No RBC transfusion group^1^	RBC transfusion group^1^	HR (95% CI)	*P* value	*P* for interaction
Lowest hemoglobin levels					0.949
< 7 g/dl	42/122 (34.4)	54/241 (22.4)	0.58 (0.39, 0.88)	0.009	
7 g/dl − 8 g/dl	101/428 (23.6)	59/366 (16.1)	0.65 (0.47, 0.90)	0.009	
8 g/dl − 9 g/dl	122/440 (27.7)	66/370 (17.8)	0.60 (0.44, 0.81)	0.001	
≥ 9 g/dl	101/301 (33.6)	68/314 (21.7)	0.58 (0.43, 0.79)	< 0.001	

^1^no. of events / total no. (%)

## Associations between different RBC volume strata and 28-day mortality

To assess the relationship between different RBC volumes and the primary outcome, the cumulative RBC volume during ICU admission was categorized into four groups: no exposure, <400 ml, <800 and ≥ 400 ml, and ≥ 800 ml. In comparison to the No RBC transfusion group, the transfusion of RBC at low(dose < 400 ml, HR: 0.60, 95% CI: 0.48, 0.75, *p* < 0.001), moderate(dose<800 and ≥ 400 ml, HR: 0.57, 95% CI: 0.44, 0.75, *p* < 0.001) and high volumes(dose ≥ 800 ml, HR: 0.71,95% CI: 0.56, 0.90, *p* = 0.004) were associated with a decreased risk of mortality(Table 4).

**Table 4:** Associations between different RBC volume strata and 28-day mortality.

RBC volume strata	No. of patients	HR	95% CI	*p*-value
< 400 ml	540	0.60	0.48, 0.75	< 0.001
400 ml-800 ml	346	0.57	0.44, 0.75	< 0.001
≥ 800 ml	405	0.71	0.56, 0.90	0.004

### Subgroup analysis

**Fig. 4:**
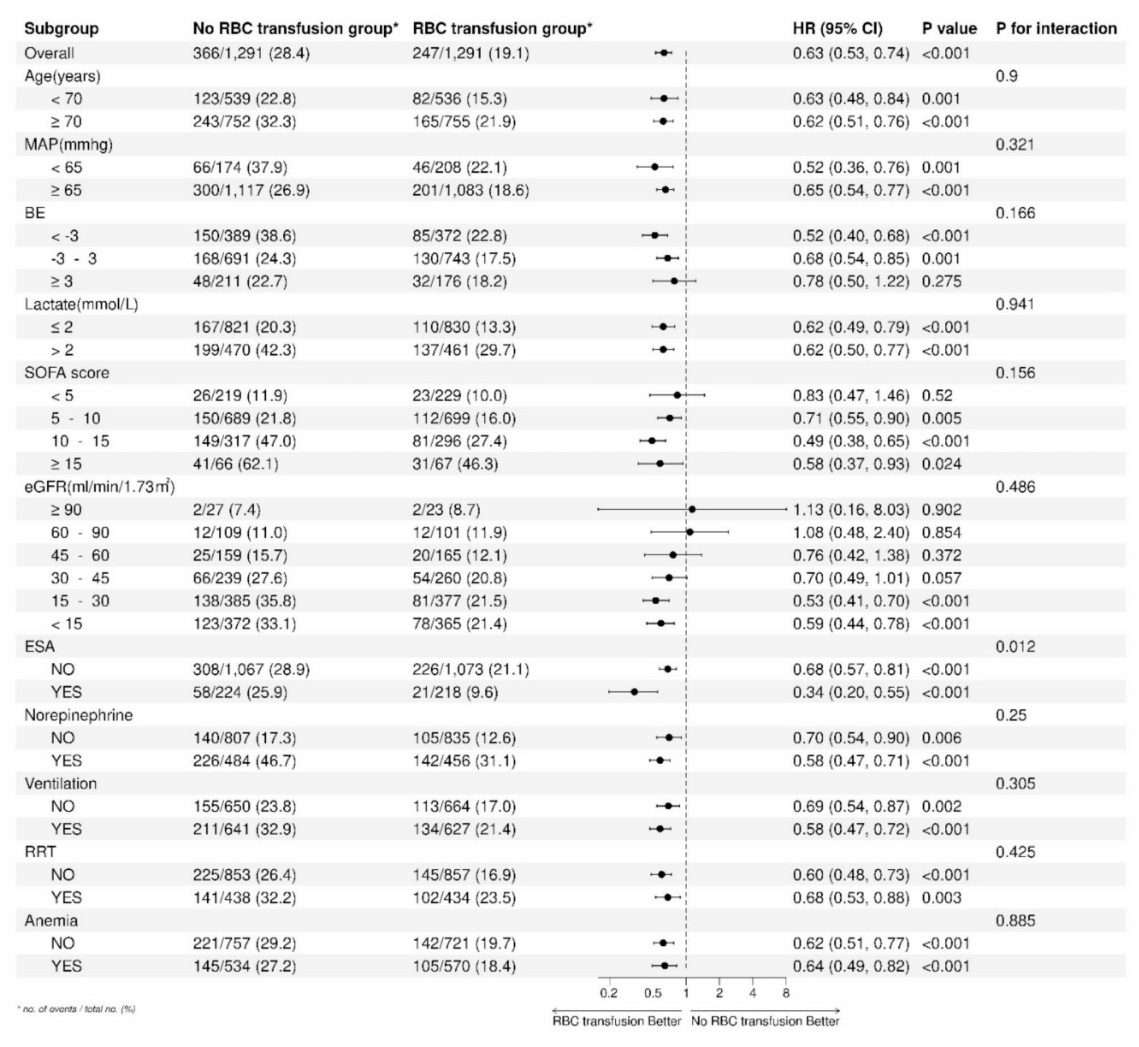
Subgroup analyses and interaction terms for 28-day mortality.

In patients with a MAP less than 65 mmHg (HR: 0.52, 95% CI: 0.36–0.76) and those receiving ESA (HR: 0.34, 95% CI: 0.20–0.55), the administration of RBC transfusion was associated with a lower 28-day mortality rate, while no interaction was detected. We found that significant improvement in outcomes occurred only when RBC transfusion was initiated in patients with an eGFR of less than 30. Furthermore, the study revealed that maintaining a SOFA score ≥ 5 and a BE value < 3 could be appropriate indications for initiating RBC transfusion in septic patients with CKD (Fig. 4).

## Discussion

Our study included 6,604 patients with sepsis and CKD. The results indicated an association between RBC transfusion and a reduced risk of 28-day mortality. Furthermore, our observations indicated that the optimal efficacy of RBC transfusion was observed in patients with eGFR less than 30, a BE less than 3, and a SOFA score greater than or equal to 5.

In patients receiving RBC transfusion, while there was a lower 28-day mortality rate, both the median ICU and hospital lengths of stay were longer. This longer stay might be due to the increased survival rate in the RBC transfusion group, leading to extended treatment and recovery time for those who survive.

Impaired renal function results in reduced urine output and a constrained overall fluid output from the body^27^. If the input requirements surpass the output, a prompt occurrence of high-volume overload occurs. Furthermore, with the improvement of the septic condition, there is a gradual enhancement in vascular tone and permeability, leading to the stepwise reabsorption of retained fluids into the bloodstream, ultimately causing high-volume overload^28^. Some earlier studies suggest that excessive fluid administration and volume overload are linked to complications in the respiratory system, infections, increased requirements and duration of mechanical ventilation, and an overall heightened risk of mortality^29–31^. Additionally, volume overload and systemic venous congestion can diminish renal perfusion, resulting in acute kidney injury^32^. Although RBC transfusion has been shown in various studies to be associated with adverse reactions such as blood volume overload, hyperkalemia, iron overload, transfusion-related infections, fever, and alloimmunization^20^, our experimental findings suggest that a low, moderate, or large volume of RBC transfusion can improve patient outcomes. Therefore, while red blood cell transfusion may lead to fluid overload for patients with sepsis and CKD, it can still improve patient outcomes. Additionally, we observed that when the eGFR was greater than or equal to 30, RBC transfusion did not reduce the 28-day mortality rate. We speculate that the improvement in mortality rates with red blood cell transfusion may be influenced by the severity of renal dysfunction in patients. Thus, special attention should be given to the eGFR levels of patients in this population.

The TRICC randomized the study cohort into a liberal transfusion group (hemoglobin threshold < 10.0 g/dL) and a restrictive transfusion group (hemoglobin threshold < 7.0 g/dL). The results revealed no statistically significant difference in the 30-day mortality rate between the two groups^21^. Likewise, in the TRISS study, when comparing the restrictive transfusion group with the liberal transfusion group, no statistically significant differences were observed in terms of RBC transfusion volume, 90-day mortality, or rates of transfusion-related adverse reactions^22^. During overextended follow-up, there was no statistically significant difference in the 1-year survival rates and Health-related Quality of Life (HRQOL) between the two groups^33^. However, both of these extensive randomized controlled trial (RCT) studies were limited by including insufficient individuals predisposed to anemia. Consequently, this study specifically enrolled patients with sepsis and CKD. Our study also evaluated whether hemoglobin levels would affect the therapeutic effect of red blood cell transfusion. The results indicate that regardless of the hemoglobin levels (as a continuous variable), there was no significant difference in the improvement of 28-day mortality with red blood cell transfusion (P for interaction = 0.949). This suggests that our study may have arrived at similar conclusions. Specifically, adopting a restrictive RBC transfusion strategy in patients with septic shock does not appear to influence short-term and long-term prognosis while simultaneously preserving valuable blood resources. Future large-scale RCT studies may be imperative to clarify further the safety of implementing lower RBC transfusion thresholds for critically ill and anemia-prone patients.

This study has several limitations. First, it is a retrospective investigation based on a database. It is inherently susceptible to drawbacks such as missing data and outliers, making it challenging to mitigate the inherent limitations of observational studies^34^. Second, the extensive period of the data collected from the MIMIC-IV database may have resulted in potential inaccuracies in our study results due to changes in the relevant guidelines. Third, the study cohort originated from a septic population at a single medical center and lacked geographical diversity, which may impact the generalizability of the findings due to the unique characteristics of a single-center hospital. Fourth, our study only accounted for RBC transfusion during patients’ ICU admissions. Patients in the No RBC transfusion group may have received RBC transfusions before or after their ICU admissions, which led to confounding results. Fifth, our study is a retrospective analysis based on the MIMIC-IV database, which limits our ability to compare differences in adverse reactions between the two groups post-RBC transfusion. Last, given the potential influence of RBC transfusion on the long-term outcomes of CKD patients undergoing kidney transplantation^35^, despite the lower one-year mortality rate observed in the RBC group, a comprehensive assessment of more detailed long-term outcomes remains elusive.

Our study found a significant link between RBC transfusion and reduced 28-day mortality in septic patients with CKD. However, its generalizability is limited. The data, drawn from a single U.S. academic medical center, may not reflect practices or patient demographics at community hospitals, international settings, or centers with different protocols. The focus on septic patients with CKD may not apply to those with varying kidney conditions or without CKD. Differences in transfusion practices across institutions and regions further limit the results’ applicability. As a retrospective study, it also carries the inherent limitations of historical data and potential confounders.

## Conclusions

In conclusion, RBC transfusion has the potential to increase the 28-day survival rate among septic patients with concurrent CKD. Within specific subgroups, factors such as BE value, SOFA score, and eGFR significantly impact treatment outcomes. Therefore, these variables should be taken into consideration when deciding upon the initial RBC transfusion. Further research is needed to evaluate these findings in randomized clinical trials.

## Electronic supplementary material

Below is the link to the electronic supplementary material.

Supplementary Material 1

Supplementary Material 2

Supplementary Material 3

Supplementary Material 4

Supplementary Material 5

Supplementary Material 6

## Data Availability

We explored publicly available datasets accessible through https://physionet.org/content/mimiciv.
