# Supplementary material for: Association between red blood cells transfusion and 28-day mortality rate in septic patients with concomitant chronic kidney disease
Source: Sci Rep. 2024 Oct 10;14:23769. doi: 10.1038/s41598-024-75643-3 (PMC11466974; doi:10.1038/s41598-024-75643-3)
Supplement: Supplementary file 1 — Supplementary Material 1 [file 41598_2024_75643_MOESM1_ESM.docx]

**Table S1. Patient demographics and baseline characteristics.**

| **Characteristics** | **Unmatched** | | | **Matched** | | |
| --- | --- | --- | --- | --- | --- | --- |
|  | **No RBC transfusion group**  (N = 4,067) | **RBC transfusion group**  (N = 2,537) | **p-value** | **No RBC transfusion group**  (N = 1,291) | **RBC transfusion group**,  (N = 1,291) | **p-value** |
| Age,years | 74 (64, 83) | 71 (61, 81) | <0.001 | 72 (62, 81) | 73 (63, 82) | 0.497 |
| Sex,male,No.(%) | 2,470 (61%) | 1,548 (61%) | 0.818 | 795 (62%) | 777 (60%) | 0.468 |
| ICU_type,No.(%) |  |  | <0.001 |  |  | 0.994 |
| MICU | 1,383 (34%) | 677 (27%) |  | 394 (31%) | 386 (30%) |  |
| SICU | 440 (11%) | 353 (14%) |  | 157 (12%) | 161 (12%) |  |
| CCU | 670 (16%) | 288 (11%) |  | 169 (13%) | 172 (13%) |  |
| TICU | 427 (10%) | 589 (23%) |  | 251 (19%) | 247 (19%) |  |
| Other | 1,147 (28%) | 630 (25%) |  | 320 (25%) | 325 (25%) |  |
| HR,beats/min | 86 (74, 101) | 86 (75, 100) | 0.752 | 86 (74, 100) | 86 (73, 100) | 0.749 |
| SBP,mmhg | 121 (104, 141) | 116 (101, 136) | <0.001 | 118 (103, 137) | 118 (102, 139) | 0.925 |
| DBP,mmhg | 63 (53, 76) | 59 (50, 71) | <0.001 | 60 (51, 72) | 60 (51, 73) | 0.832 |
| MAP,mmhg | 83 (72, 96) | 78 (68, 91) | <0.001 | 80 (70, 92) | 79 (69, 93) | 0.756 |
| RR,beats/min | 19.0 (16.0, 24.0) | 18.0 (15.0, 23.0) | <0.001 | 18.0 (15.0, 23.0) | 18.0 (15.0, 23.0) | 0.810 |
| Temperature,℃ | 36.72 (36.39, 37.06) | 36.61 (36.22, 37.00) | <0.001 | 36.67 (36.33, 37.06) | 36.67 (36.28, 37.06) | 0.496 |
| SPO_2_,(%) | 98.0 (95.0, 100.0) | 99.0 (96.0, 100.0) | <0.001 | 99.0 (96.0, 100.0) | 98.0 (96.0, 100.0) | 0.243 |
| WBC,10^9/L | 11 (8, 15) | 11 (8, 16) | 0.710 | 11 (8, 16) | 11 (8, 16) | 0.686 |
| Platelets,10^9/L | 199 (143, 269) | 180 (117, 260) | <0.001 | 187 (130, 272) | 193 (131, 267) | 0.962 |
| Hemoglobin,g/dL | 10.40 (9.10, 11.80) | 8.50 (7.50, 9.80) | <0.001 | 9.10 (8.30, 10.30) | 9.00 (8.10, 10.25) | 0.051 |
| Lowest hemoglobin levels,g/dL | 9.20 (8.30, 10.40) | 7.50 (6.80, 8.30) | <0.001 | 8.20 (7.50, 8.90) | 8.00 (7.20, 8.90) | 0.008 |
| Bilirubin,mg/dL | 0.60 (0.40, 1.00) | 0.70 (0.40, 1.30) | <0.001 | 0.61 (0.40, 1.04) | 0.60 (0.40, 1.10) | 0.923 |
| AST,U/L | 40 (26, 75) | 42 (25, 81) | 0.175 | 42 (27, 80) | 41 (25, 77) | 0.298 |
| ALT,U/L | 27 (17, 54) | 25 (15, 50) | <0.001 | 27 (17, 56) | 26 (16, 50) | 0.044 |
| Creatinine,mg/dL | 2.30 (1.50, 4.10) | 2.40 (1.50, 4.00) | 0.741 | 2.40 (1.50, 4.10) | 2.40 (1.50, 4.00) | 0.987 |
| BUN,mmol/L | 39 (27, 60) | 43 (28, 67) | <0.001 | 40 (27, 62) | 41 (28, 62) | 0.276 |
| pH | 7.36 (7.30, 7.40) | 7.36 (7.30, 7.42) | <0.001 | 7.36 (7.30, 7.42) | 7.36 (7.30, 7.41) | 0.555 |
| PaO_2_,mmhg | 82 (54, 134) | 105 (64, 226) | <0.001 | 92 (55, 200) | 100 (62, 189) | 0.206 |
| PaCO_2_,mmhg | 43 (37, 50) | 41 (35, 47) | <0.001 | 42 (36, 48) | 42 (36, 48) | 0.892 |
| Bicarbonate,mmhg | 23.0 (20.0, 26.0) | 22.0 (19.0, 25.0) | <0.001 | 22.0 (19.0, 26.0) | 23.0 (19.0, 26.0) | 0.660 |
| BE,mmol/L | -1.0 (-4.0, 1.0) | -1.0 (-4.3, 1.0) | <0.001 | -1.0 (-4.0, 1.0) | -1.0 (-4.0, 0.8) | 0.678 |
| Lactate,mmol/L | 1.80 (1.30, 2.50) | 1.70 (1.20, 2.57) | <0.001 | 1.70 (1.20, 2.50) | 1.70 (1.20, 2.40) | 0.963 |
| Potassium,mmol/L | 4.50 (4.00, 5.10) | 4.50 (4.00, 5.00) | 0.233 | 4.50 (4.00, 5.10) | 4.40 (4.00, 5.00) | 0.897 |
| Sodium,mmol/L | 138.0 (135.0, 141.0) | 138.0 (135.0, 141.0) | 0.628 | 138.0 (134.0, 141.0) | 138.0 (135.0, 141.0) | 0.246 |
| Chlorine,mmol/L | 101 (96, 106) | 103 (97, 108) | <0.001 | 102 (97, 108) | 103 (97, 107) | 0.374 |
| SOFA score | 6.0 (5.0, 9.0) | 8.0 (6.0, 12.0) | <0.001 | 8.0 (5.0, 10.0) | 7.0 (5.0, 10.0) | 0.474 |
| SIRS score | 3.00 (2.00, 3.00) | 3.00 (2.00, 3.00) | <0.001 | 3.00 (2.00, 3.00) | 3.00 (2.00, 3.00) | 0.563 |
| OASIS score | 34 (28, 40) | 37 (30, 44) | <0.001 | 36 (29, 42) | 35 (29, 42) | 0.468 |
| APS Ⅲ score | 56 (46, 70) | 65 (51, 85) | <0.001 | 60 (48, 78) | 60 (48, 76) | 0.557 |
| SAPS Ⅱ score | 42 (34, 50) | 45 (37, 55) | <0.001 | 44 (36, 52) | 43 (36, 52) | 0.724 |
| GCS score | 15.00 (14.00, 15.00) | 15.00 (15.00, 15.00) | <0.001 | 15.00 (15.00, 15.00) | 15.00 (15.00, 15.00) | 0.533 |
| ESA,No.(%) | 564 (14%) | 468 (18%) | <0.001 | 224 (17%) | 218 (17%) | 0.754 |
| Iron preparation,No.(%) | 516 (13%) | 370 (15%) | 0.028 | 191 (15%) | 198 (15%) | 0.700 |
| Norepinephrine,No.(%) | 1,172 (29%) | 1,151 (45%) | <0.001 | 484 (37%) | 456 (35%) | 0.252 |
| Vasopressin,No.(%) | 291 (7%) | 537 (21%) | <0.001 | 163 (13%) | 174 (13%) | 0.520 |
| Phenylephrine | 751 (18%) | 954 (38%) | <0.001 | 405 (31%) | 387 (30%) | 0.442 |
| Epinephrine | 137 (3%) | 311 (12%) | <0.001 | 91 (7%) | 90 (7%) | 0.939 |
| Ventilation,No.(%) | 1,401 (34%) | 1,568 (62%) | <0.001 | 641 (50%) | 627 (49%) | 0.582 |
| RRT,No.(%) | 1,162 (29%) | 1,017 (40%) | <0.001 | 438 (34%) | 434 (34%) | 0.868 |
| Anemia,No.(%) | 1,688 (42%) | 993 (39%) | 0.057 | 534 (41%) | 570 (44%) | 0.152 |
| eGFR,ml/min/1.73m^2^ | 26 (13, 42) | 25 (14, 43) | 0.292 | 25 (13, 42) | 25 (13, 42) | 0.726 |
