# Supplementary material for: Association between red blood cells transfusion and 28-day mortality rate in septic patients with concomitant chronic kidney disease
Source: Sci Rep. 2024 Oct 10;14:23769. doi: 10.1038/s41598-024-75643-3 (PMC11466974; doi:10.1038/s41598-024-75643-3)
Supplement: Supplementary file 2 — Supplementary Material 2 [file 41598_2024_75643_MOESM2_ESM.docx]

**Table S2. Univariate and multivariate Cox regression analysis of influencing factors before propensity score matching.**

|  | Univariate | | | Multivariate | | |
| --- | --- | --- | --- | --- | --- | --- |
| **Characteristic** | **HR** | **95% CI** | **p-value** | **HR** | **95% CI** | **p-value** |
| RBC transfusion | 0.88 | 0.79, 0.98 | 0.021 | 0.61 | 0.54, 0.70 | <0.001 |
| Age | 1.03 | 1.02, 1.03 | <0.001 | 1.03 | 1.02, 1.03 | <0.001 |
| Sex |  |  |  |  |  |  |
| Male | — | — |  |  |  |  |
| Female | 1.02 | 0.92, 1.13 | 0.689 |  |  |  |
| ICU_type |  |  |  |  |  |  |
| MICU | — | — |  | — | — |  |
| SICU | 0.68 | 0.57, 0.82 | <0.001 | 0.78 | 0.65, 0.94 | 0.009 |
| CCU | 0.99 | 0.85, 1.15 | 0.861 | 1.02 | 0.88, 1.20 | 0.766 |
| TICU | 0.34 | 0.27, 0.42 | <0.001 | 0.39 | 0.30, 0.50 | <0.001 |
| Other | 0.96 | 0.85, 1.09 | 0.545 | 0.94 | 0.83, 1.07 | 0.333 |
| HR | 1.01 | 1.00, 1.01 | <0.001 | 1.00 | 1.00, 1.00 | 0.261 |
| SBP | 0.99 | 0.99, 0.99 | <0.001 | 0.99 | 0.99, 1.00 | 0.003 |
| DBP | 1.00 | 1.00, 1.00 | 0.426 |  |  |  |
| MAP | 0.99 | 0.99, 1.00 | <0.001 | 1.01 | 1.00, 1.01 | 0.003 |
| RR | 1.04 | 1.03, 1.05 | <0.001 | 1.01 | 1.00, 1.02 | 0.002 |
| Temperature | 0.85 | 0.80, 0.89 | <0.001 | 0.94 | 0.89, 0.99 | 0.013 |
| SPO_2_ | 0.97 | 0.96, 0.98 | <0.001 | 0.99 | 0.98, 1.01 | 0.338 |
| WBC | 1.01 | 1.00, 1.01 | <0.001 | 1.00 | 1.00, 1.01 | 0.300 |
| Platelets | 1.00 | 1.00, 1.00 | 0.009 | 1.00 | 1.00, 1.00 | 0.041 |
| Hemoglobin | 1.02 | 0.99, 1.04 | 0.216 |  |  |  |
| Lowest hemoglobin levels | 1.01 | 0.99, 1.04 | 0.326 | 1.05 | 0.99, 1.11 | 0.105 |
| Creatinine | 0.98 | 0.96, 1.01 | 0.145 |  |  |  |
| Bilirubin | 1.04 | 1.03, 1.05 | <0.001 | 1.02 | 1.01, 1.03 | 0.003 |
| AST | 1.00 | 1.00, 1.00 | 0.002 | 1.00 | 1.00, 1.00 | 0.494 |
| ALT | 1.00 | 1.00, 1.00 | <0.001 | 1.00 | 1.00, 1.00 | 0.975 |
| BUN | 1.01 | 1.00, 1.01 | <0.001 | 1.00 | 1.00, 1.00 | 0.359 |
| pH | 0.19 | 0.12, 0.31 | <0.001 | 1.83 | 0.92, 3.63 | 0.083 |
| PO_2_ | 1.00 | 1.00, 1.00 | <0.001 | 1.00 | 1.00, 1.00 | 0.002 |
| PCO_2_ | 1.00 | 1.00, 1.00 | 0.769 |  |  |  |
| Bicarbonate | 0.98 | 0.97, 0.98 | <0.001 | 1.00 | 0.98, 1.01 | 0.831 |
| BE | 0.99 | 0.99, 1.00 | <0.001 | 1.01 | 0.99, 1.02 | 0.486 |
| Lactate | 1.14 | 1.12, 1.16 | <0.001 | 1.06 | 1.04, 1.09 | <0.001 |
| Potassium | 1.02 | 0.97, 1.07 | 0.416 |  |  |  |
| Sodium | 1.02 | 1.01, 1.03 | <0.001 | 1.02 | 1.01, 1.02 | <0.001 |
| Chlorine | 1.00 | 0.99, 1.01 | 0.912 |  |  |  |
| SOFA score | 1.08 | 1.07, 1.10 | <0.001 | 1.00 | 0.98, 1.03 | 0.825 |
| SIRS score | 1.33 | 1.26, 1.41 | <0.001 | 1.07 | 1.00, 1.14 | 0.063 |
| OASIS score | 1.06 | 1.05, 1.07 | <0.001 | 1.00 | 0.99, 1.01 | 0.849 |
| APS Ⅲ score | 1.03 | 1.03, 1.03 | <0.001 | 1.02 | 1.02, 1.02 | <0.001 |
| SAPS Ⅱ score | 1.04 | 1.04, 1.05 | <0.001 | 1.00 | 1.00, 1.01 | 0.093 |
| GCS score | 0.94 | 0.92, 0.95 | <0.001 | 0.99 | 0.97, 1.01 | 0.381 |
| ESA | 0.57 | 0.49, 0.68 | <0.001 | 0.55 | 0.45, 0.66 | <0.001 |
| Iron preparation | 0.76 | 0.65, 0.89 | <0.001 | 0.83 | 0.71, 0.99 | 0.033 |
| Norepinephrine | 2.62 | 2.37, 2.90 | <0.001 | 1.37 | 1.19, 1.58 | <0.001 |
| Vasopressin | 3.31 | 2.96, 3.71 | <0.001 | 1.71 | 1.47, 1.99 | <0.001 |
| Phenylephrine | 1.38 | 1.23, 1.53 | <0.001 | 1.01 | 0.89, 1.15 | 0.872 |
| Epinephrine | 1.45 | 1.21, 1.74 | <0.001 | 1.27 | 1.03, 1.56 | 0.025 |
| Ventilation | 1.50 | 1.35, 1.66 | <0.001 | 1.04 | 0.89, 1.22 | 0.628 |
| RRT | 1.15 | 1.03, 1.27 | 0.012 | 0.98 | 0.85, 1.13 | 0.775 |
| Anemia | 0.88 | 0.79, 0.97 | 0.014 | 0.89 | 0.79, 0.99 | 0.030 |
| eGFR | 0.99 | 0.99, 0.99 | <0.001 | 0.99 | 0.99, 1.00 | <0.001 |
