# Supplementary material for: Association between red blood cells transfusion and 28-day mortality rate in septic patients with concomitant chronic kidney disease
Source: Sci Rep. 2024 Oct 10;14:23769. doi: 10.1038/s41598-024-75643-3 (PMC11466974; doi:10.1038/s41598-024-75643-3)
Supplement: Supplementary file 3 — Supplementary Material 3 [file 41598_2024_75643_MOESM3_ESM.docx]

**Table S3. Univariate and multivariate cox regression analysis of influencing factors after propensity score matching.**

|  | Univariate | | | Multivariate | | |
| --- | --- | --- | --- | --- | --- | --- |
| **Characteristic** | **HR** | **95% CI** | **p-value** | **HR** | **95% CI** | **p-value** |
| RBC transfusion | 0.63 | 0.53, 0.74 | <0.001 | 0.60 | 0.51, 0.71 | <0.001 |
| Age | 1.02 | 1.01, 1.03 | <0.001 | 1.02 | 1.01, 1.03 | <0.001 |
| Sex |  |  |  |  |  |  |
| Male | — | — |  |  |  |  |
| Female | 1.02 | 0.87, 1.20 | 0.797 |  |  |  |
| ICU_type |  |  |  |  |  |  |
| MICU | — | — |  |  |  |  |
| SICU | 0.25 | 0.18, 0.36 | <0.001 | 0.93 | 0.71, 1.23 | 0.625 |
| CCU | 0.95 | 0.75, 1.19 | 0.651 | 1.09 | 0.85, 1.40 | 0.473 |
| TICU | 0.72 | 0.56, 0.93 | 0.010 | 0.39 | 0.27, 0.56 | <0.001 |
| Other | 0.68 | 0.51, 0.92 | 0.013 | 0.76 | 0.62, 0.94 | 0.012 |
| HR | 1.00 | 1.00, 1.01 | 0.021 | 1.00 | 0.99, 1.00 | 0.100 |
| SBP | 0.99 | 0.99, 0.99 | <0.001 | 1.00 | 0.99, 1.00 | 0.137 |
| DBP | 1.00 | 0.99, 1.00 | 0.219 |  |  |  |
| MAP | 0.99 | 0.99, 1.00 | <0.001 | 1.00 | 0.99, 1.01 | 0.467 |
| RR | 1.05 | 1.04, 1.06 | <0.001 | 1.02 | 1.01, 1.03 | 0.008 |
| Temperature | 0.84 | 0.77, 0.91 | <0.001 | 0.92 | 0.85, 1.00 | 0.038 |
| SPO_2_ | 0.98 | 0.97, 0.99 | 0.001 | 1.01 | 0.99, 1.03 | 0.362 |
| WBC | 1.01 | 1.00, 1.01 | <0.001 | 1.00 | 1.00, 1.01 | 0.108 |
| Platelets | 1.00 | 1.00, 1.00 | 0.067 | 1.00 | 1.00, 1.00 | 0.013 |
| Hemoglobin | 1.07 | 1.02, 1.12 | 0.003 | 0.98 | 0.92, 1.05 | 0.623 |
| Lowest hemoglobin levels | 1.06 | 1.00, 1.13 | 0.051 | 0.96 | 0.87, 1.06 | 0.436 |
| Creatinine | 1.04 | 1.01, 1.07 | 0.019 | 0.98 | 0.93, 1.04 | 0.482 |
| Bilirubin | 1.04 | 1.02, 1.05 | <0.001 | 1.01 | 0.99, 1.03 | 0.214 |
| AST | 1.00 | 1.00, 1.00 | <0.001 | 1.00 | 1.00, 1.00 | 0.347 |
| ALT | 1.00 | 1.00, 1.00 | <0.001 | 1.00 | 1.00, 1.00 | 0.405 |
| BUN | 1.01 | 1.00, 1.01 | <0.001 | 1.00 | 0.99, 1.00 | 0.268 |
| pH | 0.08 | 0.04, 0.17 | <0.001 | 1.53 | 0.11, 22.10 | 0.754 |
| PO2 | 1.00 | 1.00, 1.00 | <0.001 | 1.00 | 1.00, 1.00 | 0.032 |
| PCO2 | 1.00 | 1.00, 1.01 | 0.090 | 1.01 | 0.99, 1.02 | 0.492 |
| Bicarbonate | 0.97 | 0.96, 0.98 | <0.001 | 0.97 | 0.94, 1.00 | 0.054 |
| BE | 0.96 | 0.94, 0.97 | <0.001 | 1.01 | 0.96, 1.06 | 0.709 |
| Lactate | 1.16 | 1.14, 1.19 | <0.001 | 1.07 | 1.02, 1.11 | 0.004 |
| Potassium | 1.06 | 0.98, 1.15 | 0.139 |  |  |  |
| Sodium | 1.01 | 1.00, 1.03 | 0.061 | 1.04 | 1.01, 1.06 | 0.006 |
| Chlorine | 0.99 | 0.98, 1.00 | 0.010 | 0.98 | 0.95, 1.00 | 0.034 |
| SOFA score | 1.18 | 1.15, 1.20 | <0.001 | 0.98 | 0.94, 1.02 | 0.339 |
| SIRS score | 1.37 | 1.25, 1.50 | <0.001 | 1.13 | 1.01, 1.25 | 0.026 |
| OASIS score | 1.06 | 1.05, 1.07 | <0.001 | 1.00 | 0.99, 1.02 | 0.924 |
| APS Ⅲ score | 1.03 | 1.03, 1.03 | <0.001 | 1.02 | 1.01, 1.02 | <0.001 |
| SAPS Ⅱ score | 1.04 | 1.04, 1.05 | <0.001 | 1.00 | 0.99, 1.01 | 0.581 |
| GCS score | 0.95 | 0.92, 0.98 | 0.003 | 0.97 | 0.94, 1.01 | 0.195 |
| ESA | 0.66 | 0.52, 0.84 | <0.001 | 0.50 | 0.39, 0.66 | <0.001 |
| Iron preparation | 0.72 | 0.56, 0.92 | 0.009 | 0.82 | 0.63, 1.05 | 0.116 |
| Norepinephrine | 3.11 | 2.65, 3.66 | <0.001 | 1.41 | 1.13, 1.77 | 0.002 |
| Vasopressin | 3.90 | 3.28, 4.63 | <0.001 | 1.89 | 1.52, 2.37 | <0.001 |
| Phenylephrine | 1.26 | 1.07, 1.49 | 0.007 | 1.04 | 0.85, 1.27 | 0.713 |
| Epinephrine | 1.61 | 1.24, 2.11 | <0.001 | 1.52 | 1.12, 2.06 | 0.007 |
| Ventilation | 1.42 | 1.21, 1.67 | <0.001 | 1.07 | 0.84, 1.36 | 0.573 |
| RRT | 1.33 | 1.13, 1.56 | <0.001 | 1.04 | 0.83, 1.30 | 0.759 |
| Anemia | 0.90 | 0.76, 1.05 | 0.186 |  |  |  |
| eGFR | 0.99 | 0.98, 0.99 | <0.001 | 0.99 | 0.98, 1.00 | 0.011 |
