# Supplementary material for: Association between red blood cells transfusion and 28-day mortality rate in septic patients with concomitant chronic kidney disease
Source: Sci Rep. 2024 Oct 10;14:23769. doi: 10.1038/s41598-024-75643-3 (PMC11466974; doi:10.1038/s41598-024-75643-3)
Supplement: Supplementary file 4 — Supplementary Material 4 [file 41598_2024_75643_MOESM4_ESM.docx]

**Table S4. Percentage of missing data from primary cohort.**

| Variables | n=6,604 |
| --- | --- |
| Age | 0% |
| Sex | 0% |
| ICU type | 0% |
| HR | 0.01% |
| SBP | 0.01% |
| DBP | 0.02% |
| MAP | 0.02% |
| RR | 0.01% |
| Temperature | 0.37% |
| SPO_2_ | 0.06% |
| WBC | 0.01% |
| Platelets | 0.01% |
| Hemoglobin | 0.01% |
| Lowest hemoglobin levels | 0.01% |
| Bilirubin | 0.01% |
| AST | 24.61% |
| ALT | 23.47% |
| Creatinine | 0.37% |
| BUN | 0.01% |
| pH | 32.43.% |
| PaO_2_ | 27.65% |
| PaCO_2_ | 27.65% |
| Bicarbonate | 0.01% |
| BE | 27.65% |
| Lactate | 27.09% |
| Potassium | 0.01% |
| Sodium | 0.01% |
| Chlorine | 0.01% |
| SOFA score | 0% |
| SIRS score | 0% |
| OASIS score | 0% |
| APSⅢ score | 0% |
| SAPSⅡ score | 0% |
| GCS score | 0% |
| ESA | 0% |
| Iron preparation | 0% |
| Norepinephrine | 0% |
| Vasopressin | 0% |
| Phenylephrine | 0% |
| Epinephrine | 0% |
| Ventilation | 0% |
| RRT | 0% |
| Anemia | 0% |
| eGFR | 0% |
