# Supplementary figures and images for: Association between red blood cells transfusion and 28-day mortality rate in septic patients with concomitant chronic kidney disease
Source: Sci Rep. 2024 Oct 10;14:23769. doi: 10.1038/s41598-024-75643-3 (PMC11466974; doi:10.1038/s41598-024-75643-3)

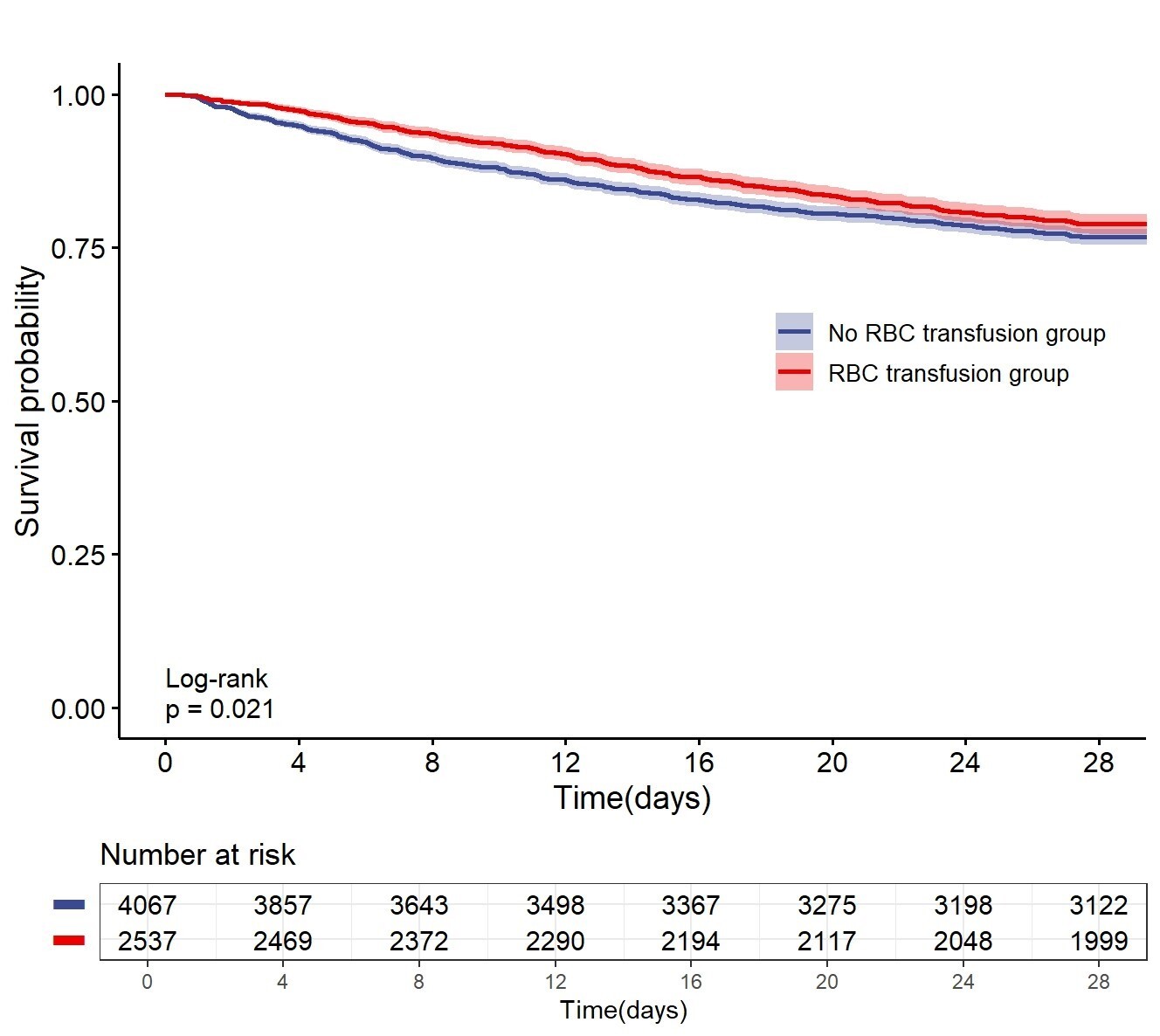

Supplement: Supplementary file 5 — Supplementary Material 5 [file 41598_2024_75643_MOESM5_ESM.jpeg]
