# Supplementary material for: Association between red blood cells transfusion and 28-day mortality rate in septic patients with concomitant chronic kidney disease
Source: Sci Rep. 2024 Oct 10;14:23769. doi: 10.1038/s41598-024-75643-3 (PMC11466974; doi:10.1038/s41598-024-75643-3)

Table S1. Patient demographics and baseline characteristics.

Characteristics	Unmatched			Matched		
	No RBC transfusion group (N = 4,067)	RBC transfusion group (N = 2,537)	p-value	No RBC transfusion group (N = 1,291)	RBC transfusion group, (N = 1,291)	p-value
Age,years	74 (64, 83)	71 (61, 81)	<0.001	72 (62, 81)	73 (63, 82)	0.497
Sex,male,No.(%)	2,470 (61%)	1,548 (61%)	0.818	795 (62%)	777 (60%)	0.468
ICU_type,No.(%)			<0.001			0.994
MICU	1,383 (34%)	677 (27%)		394 (31%)	386 (30%)	
SICU	440 (11%)	353 (14%)		157 (12%)	161 (12%)	
CCU	670 (16%)	288 (11%)		169 (13%)	172 (13%)	
TICU	427 (10%)	589 (23%)		251 (19%)	247 (19%)	
Other	1,147 (28%)	630 (25%)		320 (25%)	325 (25%)	
HR,beats/min	86 (74, 101)	86 (75, 100)	0.752	86 (74, 100)	86 (73, 100)	0.749
SBP,mmhg	121 (104, 141)	116 (101, 136)	<0.001	118 (103, 137)	118 (102, 139)	0.925
DBP,mmhg	63 (53, 76)	59 (50, 71)	<0.001	60 (51, 72)	60 (51, 73)	0.832
MAP,mmhg	83 (72, 96)	78 (68, 91)	<0.001	80 (70, 92)	79 (69, 93)	0.756
RR,beats/min	19.0 (16.0, 24.0)	18.0 (15.0, 23.0)	<0.001	18.0 (15.0, 23.0)	18.0 (15.0, 23.0)	0.810
Temperature,℃	36.72 (36.39, 37.06)	36.61 (36.22, 37.00)	<0.001	36.67 (36.33, 37.06)	36.67 (36.28, 37.06)	0.496
SPO_2,(%)	98.0 (95.0, 100.0)	99.0 (96.0, 100.0)	<0.001	99.0 (96.0, 100.0)	98.0 (96.0, 100.0)	0.243
WBC,10^9/L	11 (8, 15)	11 (8, 16)	0.710	11 (8, 16)	11 (8, 16)	0.686
Platelets,10^9/L	199 (143, 269)	180 (117, 260)	<0.001	187 (130, 272)	193 (131, 267)	0.962
Hemoglobin,g/dL	10.40 (9.10, 11.80)	8.50 (7.50, 9.80)	<0.001	9.10 (8.30, 10.30)	9.00 (8.10, 10.25)	0.051
Lowest hemoglobin levels,g/dL	9.20 (8.30, 10.40)	7.50 (6.80, 8.30)	<0.001	8.20 (7.50, 8.90)	8.00 (7.20, 8.90)	0.008
Bilirubin,mg/dL	0.60 (0.40, 1.00)	0.70 (0.40, 1.30)	<0.001	0.61 (0.40, 1.04)	0.60 (0.40, 1.10)	0.923
AST,U/L	40 (26, 75)	42 (25, 81)	0.175	42 (27, 80)	41 (25, 77)	0.298
ALT,U/L	27 (17, 54)	25 (15, 50)	<0.001	27 (17, 56)	26 (16, 50)	0.044
Creatinine,mg/dL	2.30 (1.50, 4.10)	2.40 (1.50, 4.00)	0.741	2.40 (1.50, 4.10)	2.40 (1.50, 4.00)	0.987
BUN,mmol/L	39 (27, 60)	43 (28, 67)	<0.001	40 (27, 62)	41 (28, 62)	0.276
pH	7.36 (7.30, 7.40)	7.36 (7.30, 7.42)	<0.001	7.36 (7.30, 7.42)	7.36 (7.30, 7.41)	0.555
PaO_2,mmhg	82 (54, 134)	105 (64, 226)	<0.001	92 (55, 200)	100 (62, 189)	0.206
$PaCO_2$,mmhg	43 (37, 50)	41 (35, 47)	<0.001	42 (36, 48)	42 (36, 48)	0.892
Bicarbonate,mmhg	23.0 (20.0, 26.0)	22.0 (19.0, 25.0)	<0.001	22.0 (19.0, 26.0)	23.0 (19.0, 26.0)	0.660
BE,mmol/L	-1.0 (-4.0, 1.0)	-1.0 (-4.3, 1.0)	<0.001	-1.0 (-4.0, 1.0)	-1.0 (-4.0, 0.8)	0.678
Lactate,mmol/L	1.80 (1.30, 2.50)	1.70 (1.20, 2.57)	<0.001	1.70 (1.20, 2.50)	1.70 (1.20, 2.40)	0.963
Potassium,mmol/L	4.50 (4.00, 5.10)	4.50 (4.00, 5.00)	0.233	4.50 (4.00, 5.10)	4.40 (4.00, 5.00)	0.897
Sodium,mmol/L	138.0 (135.0, 141.0)	138.0 (135.0, 141.0)	0.628	138.0 (134.0, 141.0)	138.0 (135.0, 141.0)	0.246
Chlorine,mmol/L	101 (96, 106)	103 (97, 108)	<0.001	102 (97, 108)	103 (97, 107)	0.374
SOFA score	6.0 (5.0, 9.0)	8.0 (6.0, 12.0)	<0.001	8.0 (5.0, 10.0)	7.0 (5.0, 10.0)	0.474
SIRS score	3.00 (2.00, 3.00)	3.00 (2.00, 3.00)	<0.001	3.00 (2.00, 3.00)	3.00 (2.00, 3.00)	0.563
OASIS score	34 (28, 40)	37 (30, 44)	<0.001	36 (29, 42)	35 (29, 42)	0.468
APS III score	56 (46, 70)	65 (51, 85)	<0.001	60 (48, 78)	60 (48, 76)	0.557
SAPS II score	42 (34, 50)	45 (37, 55)	<0.001	44 (36, 52)	43 (36, 52)	0.724
GCS score	15.00 (14.00, 15.00)	15.00 (15.00, 15.00)	<0.001	15.00 (15.00, 15.00)	15.00 (15.00, 15.00)	0.533
ESA,No.(%)	564 (14%)	468 (18%)	<0.001	224 (17%)	218 (17%)	0.754
Iron preparation,No.(%)	516 (13%)	370 (15%)	0.028	191 (15%)	198 (15%)	0.700
Norepinephrine,No.(%)	1,172 (29%)	1,151 (45%)	<0.001	484 (37%)	456 (35%)	0.252
Vasopressin,No.(%)	291 (7%)	537 (21%)	<0.001	163 (13%)	174 (13%)	0.520
Phenylephrine	751 (18%)	954 (38%)	<0.001	405 (31%)	387 (30%)	0.442
Epinephrine	137 (3%)	311 (12%)	<0.001	91 (7%)	90 (7%)	0.939
Ventilation,No.(%)	1,401 (34%)	1,568 (62%)	<0.001	641 (50%)	627 (49%)	0.582
RRT,No.(%)	1,162 (29%)	1,017 (40%)	<0.001	438 (34%)	434 (34%)	0.868
Anemia,No.(%)	1,688 (42%)	993 (39%)	0.057	534 (41%)	570 (44%)	0.152

Characteristics	Unmatched			Matched		
	No RBC transfusion group (N = 4,067)	RBC transfusion group (N = 2,537)	p-value	No RBC transfusion group (N = 1,291)	RBC transfusion group, (N = 1,291)	p-value
eGFR,ml/min/1.73m^2	26 (13, 42)	25 (14, 43)	0.292	25 (13, 42)	25 (13, 42)	0.726

Table S2. Univariate and multivariate Cox regression analysis of influencing factors before propensity score matching.

	Univariate			Multivariate		
**Characteristic**	**HR**	**95% CI**	**p-value**	**HR**	**95% CI**	**p-value**
RBC transfusion	0.88	0.79, 0.98	0.021	0.61	0.54, 0.70	<0.001
Age	1.03	1.02, 1.03	<0.001	1.03	1.02, 1.03	<0.001
Sex						
Male	—	—				
Female	1.02	0.92, 1.13	0.689			
ICU_type						
MICU	—	—		—	—	
SICU	0.68	0.57, 0.82	<0.001	0.78	0.65, 0.94	0.009
CCU	0.99	0.85, 1.15	0.861	1.02	0.88, 1.20	0.766
TICU	0.34	0.27, 0.42	<0.001	0.39	0.30, 0.50	<0.001
Other	0.96	0.85, 1.09	0.545	0.94	0.83, 1.07	0.333
HR	1.01	1.00, 1.01	<0.001	1.00	1.00, 1.00	0.261
SBP	0.99	0.99, 0.99	<0.001	0.99	0.99, 1.00	0.003
DBP	1.00	1.00, 1.00	0.426			
MAP	0.99	0.99, 1.00	<0.001	1.01	1.00, 1.01	0.003
RR	1.04	1.03, 1.05	<0.001	1.01	1.00, 1.02	0.002
Temperature	0.85	0.80, 0.89	<0.001	0.94	0.89, 0.99	0.013
SPO_2	0.97	0.96, 0.98	<0.001	0.99	0.98, 1.01	0.338
WBC	1.01	1.00, 1.01	<0.001	1.00	1.00, 1.01	0.300
Platelets	1.00	1.00, 1.00	0.009	1.00	1.00, 1.00	0.041
Hemoglobin	1.02	0.99, 1.04	0.216			
Lowest hemoglobin levels	1.01	0.99, 1.04	0.326	1.05	0.99, 1.11	0.105
Creatinine	0.98	0.96, 1.01	0.145			
Bilirubin	1.04	1.03, 1.05	<0.001	1.02	1.01, 1.03	0.003
AST	1.00	1.00, 1.00	0.002	1.00	1.00, 1.00	0.494
ALT	1.00	1.00, 1.00	<0.001	1.00	1.00, 1.00	0.975
BUN	1.01	1.00, 1.01	<0.001	1.00	1.00, 1.00	0.359
pH	0.19	0.12, 0.31	<0.001	1.83	0.92, 3.63	0.083
PO_2	1.00	1.00, 1.00	<0.001	1.00	1.00, 1.00	0.002
PCO_2	1.00	1.00, 1.00	0.769			
Bicarbonate	0.98	0.97, 0.98	<0.001	1.00	0.98, 1.01	0.831
BE	0.99	0.99, 1.00	<0.001	1.01	0.99, 1.02	0.486
Lactate	1.14	1.12, 1.16	<0.001	1.06	1.04, 1.09	<0.001
Potassium	1.02	0.97, 1.07	0.416			
Sodium	1.02	1.01, 1.03	<0.001	1.02	1.01, 1.02	<0.001
Chlorine	1.00	0.99, 1.01	0.912			
SOFA score	1.08	1.07, 1.10	<0.001	1.00	0.98, 1.03	0.825
SIRS score	1.33	1.26, 1.41	<0.001	1.07	1.00, 1.14	0.063
OASIS score	1.06	1.05, 1.07	<0.001	1.00	0.99, 1.01	0.849
APS III score	1.03	1.03, 1.03	<0.001	1.02	1.02, 1.02	<0.001
SAPS II score	1.04	1.04, 1.05	<0.001	1.00	1.00, 1.01	0.093
GCS score	0.94	0.92, 0.95	<0.001	0.99	0.97, 1.01	0.381
ESA	0.57	0.49, 0.68	<0.001	0.55	0.45, 0.66	<0.001
Iron preparation	0.76	0.65, 0.89	<0.001	0.83	0.71, 0.99	0.033
Norepinephrine	2.62	2.37, 2.90	<0.001	1.37	1.19, 1.58	<0.001
Vasopressin	3.31	2.96, 3.71	<0.001	1.71	1.47, 1.99	<0.001

	Univariate			Multivariate		
**Characteristic**	**HR**	**95% CI**	**p-value**	**HR**	**95% CI**	**p-value**
Phenylephrine	1.38	1.23, 1.53	<0.001	1.01	0.89, 1.15	0.872
Epinephrine	1.45	1.21, 1.74	<0.001	1.27	1.03, 1.56	0.025
Ventilation	1.50	1.35, 1.66	<0.001	1.04	0.89, 1.22	0.628
RRT	1.15	1.03, 1.27	0.012	0.98	0.85, 1.13	0.775
Anemia	0.88	0.79, 0.97	0.014	0.89	0.79, 0.99	0.030
eGFR	0.99	0.99, 0.99	<0.001	0.99	0.99, 1.00	<0.001

Table S3. Univariate and multivariate cox regression analysis of influencing factors after propensity score matching.

	Univariate			Multivariate		
**Characteristic**	**HR**	**95% CI**	**p-value**	**HR**	**95% CI**	**p-value**
RBC transfusion	0.63	0.53, 0.74	<0.001	0.60	0.51, 0.71	<0.001
Age	1.02	1.01, 1.03	<0.001	1.02	1.01, 1.03	<0.001
Sex						
Male	—	—				
Female	1.02	0.87, 1.20	0.797			
ICU_type						
MICU	—	—				
SICU	0.25	0.18, 0.36	<0.001	0.93	0.71, 1.23	0.625
CCU	0.95	0.75, 1.19	0.651	1.09	0.85, 1.40	0.473
TICU	0.72	0.56, 0.93	0.010	0.39	0.27, 0.56	<0.001
Other	0.68	0.51, 0.92	0.013	0.76	0.62, 0.94	0.012
HR	1.00	1.00, 1.01	0.021	1.00	0.99, 1.00	0.100
SBP	0.99	0.99, 0.99	<0.001	1.00	0.99, 1.00	0.137
DBP	1.00	0.99, 1.00	0.219			
MAP	0.99	0.99, 1.00	<0.001	1.00	0.99, 1.01	0.467
RR	1.05	1.04, 1.06	<0.001	1.02	1.01, 1.03	0.008
Temperature	0.84	0.77, 0.91	<0.001	0.92	0.85, 1.00	0.038
SPO_2	0.98	0.97, 0.99	0.001	1.01	0.99, 1.03	0.362
WBC	1.01	1.00, 1.01	<0.001	1.00	1.00, 1.01	0.108
Platelets	1.00	1.00, 1.00	0.067	1.00	1.00, 1.00	0.013
Hemoglobin	1.07	1.02, 1.12	0.003	0.98	0.92, 1.05	0.623
Lowest hemoglobin levels	1.06	1.00, 1.13	0.051	0.96	0.87, 1.06	0.436
Creatinine	1.04	1.01, 1.07	0.019	0.98	0.93, 1.04	0.482
Bilirubin	1.04	1.02, 1.05	<0.001	1.01	0.99, 1.03	0.214
AST	1.00	1.00, 1.00	<0.001	1.00	1.00, 1.00	0.347
ALT	1.00	1.00, 1.00	<0.001	1.00	1.00, 1.00	0.405
BUN	1.01	1.00, 1.01	<0.001	1.00	0.99, 1.00	0.268
pH	0.08	0.04, 0.17	<0.001	1.53	0.11, 22.10	0.754
PO2	1.00	1.00, 1.00	<0.001	1.00	1.00, 1.00	0.032
PCO2	1.00	1.00, 1.01	0.090	1.01	0.99, 1.02	0.492
Bicarbonate	0.97	0.96, 0.98	<0.001	0.97	0.94, 1.00	0.054
BE	0.96	0.94, 0.97	<0.001	1.01	0.96, 1.06	0.709
Lactate	1.16	1.14, 1.19	<0.001	1.07	1.02, 1.11	0.004
Potassium	1.06	0.98, 1.15	0.139			
Sodium	1.01	1.00, 1.03	0.061	1.04	1.01, 1.06	0.006
Chlorine	0.99	0.98, 1.00	0.010	0.98	0.95, 1.00	0.034
SOFA score	1.18	1.15, 1.20	<0.001	0.98	0.94, 1.02	0.339
SIRS score	1.37	1.25, 1.50	<0.001	1.13	1.01, 1.25	0.026
OASIS score	1.06	1.05, 1.07	<0.001	1.00	0.99, 1.02	0.924
APS III score	1.03	1.03, 1.03	<0.001	1.02	1.01, 1.02	<0.001
SAPS II score	1.04	1.04, 1.05	<0.001	1.00	0.99, 1.01	0.581
GCS score	0.95	0.92, 0.98	0.003	0.97	0.94, 1.01	0.195
ESA	0.66	0.52, 0.84	<0.001	0.50	0.39, 0.66	<0.001

	Univariate			Multivariate		
**Characteristic**	**HR**	**95% CI**	**p-value**	**HR**	**95% CI**	**p-value**
Iron preparation	0.72	0.56, 0.92	0.009	0.82	0.63, 1.05	0.116
Norepinephrine	3.11	2.65, 3.66	<0.001	1.41	1.13, 1.77	0.002
Vasopressin	3.90	3.28, 4.63	<0.001	1.89	1.52, 2.37	<0.001
Phenylephrine	1.26	1.07, 1.49	0.007	1.04	0.85, 1.27	0.713
Epinephrine	1.61	1.24, 2.11	<0.001	1.52	1.12, 2.06	0.007
Ventilation	1.42	1.21, 1.67	<0.001	1.07	0.84, 1.36	0.573
RRT	1.33	1.13, 1.56	<0.001	1.04	0.83, 1.30	0.759
Anemia	0.90	0.76, 1.05	0.186			
eGFR	0.99	0.98, 0.99	<0.001	0.99	0.98, 1.00	0.011

Table S4. Percentage of missing data from primary cohort.

Variables	n=6,604
Age	0%
Sex	0%
ICU type	0%
HR	0.01%
SBP	0.01%
DBP	0.02%
MAP	0.02%
RR	0.01%
Temperature	0.37%
SPO_2	0.06%
WBC	0.01%
Platelets	0.01%
Hemoglobin	0.01%
Lowest hemoglobin levels	0.01%
Bilirubin	0.01%
AST	24.61%
ALT	23.47%
Creatinine	0.37%
BUN	0.01%
pH	32.43.%
PaO_2	27.65%
$PaCO_2$	27.65%
Bicarbonate	0.01%
BE	27.65%
Lactate	27.09%
Potassium	0.01%
Sodium	0.01%
Chlorine	0.01%
SOFA score	0%
SIRS score	0%
OASIS score	0%
APSIII score	0%
SAPSII score	0%
GCS score	0%
ESA	0%
Iron preparation	0%
Norepinephrine	0%
Vasopressin	0%
Phenylephrine	0%
Epinephrine	0%
Ventilation	0%
RRT	0%
Anemia	0%
eGFR	0%

Fig S1. Kaplan-Meier survival analysis curves for 28-day mortality before propensity score matching.

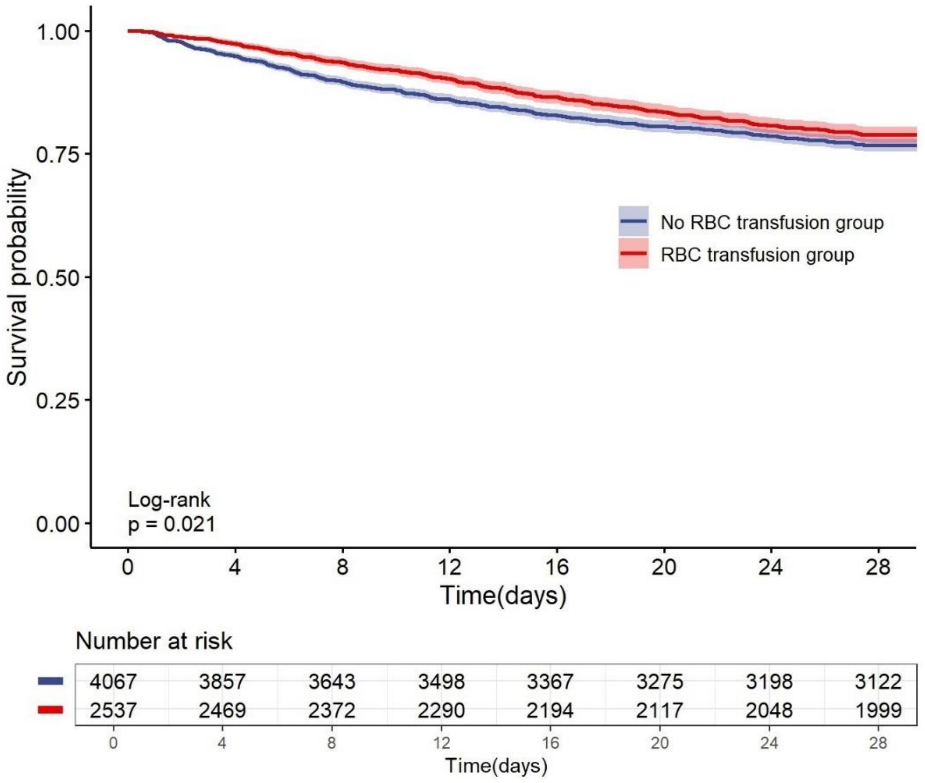

Supplement: Supplementary file 6 — Supplementary Material 6 [file 41598_2024_75643_MOESM6_ESM.pdf]
